# Cross-Strait climate change and agricultural product loss

**DOI:** 10.1007/s11356-019-05166-2

**Published:** 2019-05-22

**Authors:** Hsing-Chun Lin, Li-Chen Chou, Wan-Hao Zhang

**Affiliations:** 1 Department of Applied Economics, National Chiayi University, Chiayi, Taiwan; 2 Department of Economics, Wenzhou Business College, Wenzhou, China; 3 School of Politics and Economics, King’s College London, London, UK

**Keywords:** Climate change loss, Inter-Country Input-Output (ICIO) Model, Cross-Strait, Economic analysis

## Abstract

The structure of agricultural industries at Cross-Strait differs as climate change is considered. In fact, its influence on their agriculture and other industries vary when the impact produced by natural disasters due to climate change are faced. To estimate direct and indirect losses caused by natural disasters, this study applies Inter-Country Input-Output (ICIO) analysis developed by Miller and Blair (2009) to discuss the development among Cross-Strait industries as they face disaster losses. The data sources used in this article are from Lin (2013), Cross-Strait ICIO table, and the statistics of agriculture in the periods 2005–2017 for Taiwan and Mainland China. The main results from our ICIO analysis are as follows: the value-added losses caused by natural disasters mainly involve agriculture, forestry, fishery, wholesale and retail trade, animal feed, and chemical fertilizer industries. These sectors account for 87.4% in Mainland China and 94.6% in Taiwan of total separately.

## Introduction

Due to the impacts of warm and ocean currents, Taiwan’s climate is influenced by continental and maritime weathers. Averagely, there are three or four typhoons during summer and autumn in Taiwan. Typhoons bring abundant rainfall spatially distributed in an unequal manner. Usually, such natural disasters trigger floods, landslides, and other losses. For instance, typhoon Morakot caused more than 600 fatal casualties and 164 billion losses in crops and infrastructures in 2009. Mainland China also suffered by natural disasters such as typhoon and droughts in recent years. Though total rainfall does not change too much year by year, its distribution varies monthly. According to Office of State Flood Control and Drought Relief Headquarters, 43% of wheat fields were stricken by drought and 429 million people faced the problem of drinking water availability.

Climate change affects many fields in the environment, according to Ahmed et al. (2015), they found that 33% of farmers in the study area within Punjab Province, Pakistan, were unwilling to pay for a planned climate change adaptation programme, whereas 67% were willing to pay (WTP) for the predominant reasons being ‘impacts on agricultural production’, ‘feeling responsible for my contribution to climate change’, and ‘concern for the risk posed by climate change’. The study also found farmers who were more WTP for a climate change adaptation programme were more educated, had higher incomes, and had greater concern for climate change (Ahmed et al. 2015; McElhinney 2016). The general public’s definition of natural disasters is relatively simple. As long as it is determined that disasters occurring in the surrounding natural environment will be defined as natural disasters. However, it is necessary to carefully analyse the intermediate occurrence process from the causes of disasters to the results of disasters. For the evaluation method, it is necessary to make changes and find corresponding definitions for the characteristics of the evaluation object (Mary and Pielke 2005). In other words, climate change not only affects the environment but also affects society awareness about the environment sustainability.

Following the above, although Taiwan and Mainland China possess a different industrial structure, both suffer the consequences of natural disasters, including losses in agriculture and related industries. With the aim to evaluate direct and indirect impacts of natural disasters in agriculture between Taiwan and Mainland China, the supply-side Inter-Country Output analysis (Miller and Blair 2009) has been applied. To deal with, the Cross-Strait Inter-Country Input-Output table (Lin 2013) involving 96 sectors have been used (Appendix 1), which is actually the statistics of consequences of natural disasters in crops from the Council of Agriculture Statistics in Taiwan and Mainland China from 2005 to 2017.

The structure of this article is as follows. “Literature review” contains a literature review. In “Methodology”, a discussion regarding the supply-side Inter-Country Input-Output analysis is carried out. “Empirical analysis” describes our empirical results, and finally, “Economic impact analysis” highlights the main conclusions in this paper.

## Literature review

Agriculture is a high-impact and high-vulnerability industry. Whether it is a change in rainfall patterns or extreme weather events, it will have a serious impact on agricultural production. Therefore, in order to effectively develop agricultural adaptation strategies, the mastery of agricultural loss assessment under different rainfall intensities is important (Wang et al. 2014). In order to alleviate farmers’ burden of natural disaster losses and stabilize agriculture and rural development, the relevant literatures on agricultural natural disasters or agricultural loss assessment in the past focus on agricultural disasters and their resulting safety concept with agricultural insurance (Claassen et al. 2011; Goodwin and Rejesus 2008). Some researchers also discussed the political economy of agricultural disaster relief allocation (Chang and Zilberman 2014; Garrett et al. 2006), and considered the government’s role in agricultural disasters (Goodwin and Vado 2007).

To explore the impact derived from climate change in Agro-ecosystems, previous studies usually analyse the catastrophe risk, the losses caused by the disaster. For example, Rose et al. (1997) estimated the industrial impacts by earthquake with Regional Input-Output and linear programming. Gordon et al. (1998, 2004) estimated the transport damage by the earthquake simulation in south California and measured the direct and indirect costs of earthquake which integrated the Regional Input-Output model and the price model. Fischer et al. (2002) evaluated the development of economic system in the future via Basic Linked Systems (BLS) and Global Circulation Models (GCM). The study concluded that climate change influences all the natural ecosystems. In addition, it changes regional productivity and population growth in the long-term. Rose and Liao (2005) measured the related impacts with Computational General Equilibrium model for water supply system damage by earthquake. Yamano et al. (2007) explored the impacts on the regional industries which came from electricity and transport internet injuries caused by natural disasters in Japan; they found direct damages exceed indirect damages a lot and the impacts of manufacturing and commercial districts are worse than other areas.

Shi (2009) studied the crisis and response in a society after the natural disaster happened. Masud et al. (2017) indicated that socio-demographic factors such as gender, age, education, income, and ethnicity can greatly influence the individual’s awareness, attitude, risk perception, and knowledge of climate change issues. Other studies like Krosnick et al. (2006), Lorenzoni and Pidgeon (2006), Leiserowitz (2006), and Zahran et al. (2006) have found that the influence of global warming will affect public’s attention and the effects of climate change can also be influenced to a certain extent, by specific psychological and behavioural factors.

In the evaluation of climate change and economic growth, Furen et al. (2005) stated that climate change will affect population growth and economic development. As a consequence of rainfall decreasing, the risk of drought will rise and affect the productivity of related industries. They applied Social Accounting Matrix (SAM) and set up six situations to explore the economic impact during water restrictions. As a conclusion, food and beverage sectors will bear the worst losses in all the situations. Myles et al. (2005) evaluated the agricultural impacts of Katrina and Rita hurricanes in Mississippi. They discussed their impact on employment, commercial revenue, and labour income in short, middle, and long run. The results demonstrated that the impact of hurricanes have a significant influence in commercial revenue and labour income, even 15 years after the natural disaster occurred. Okuyama (2007) explored the advantages and disadvantages among Input-Output model, Computable General Equilibrium (CGE) model, and Social Accounting Matrix (SAM). The author noticed that a model to assess the economic impact is mainly focused on its accuracy. However, every disaster differs from others and may not exhibit the same degree of hazard. Tsuchiya et al. (2007) analysed the economic impacts of transportation system and infrastructure which faced Tokai-Tonankai earthquakes. The economic damage analysis based on Input-Output analysis and Computational General Equilibrium in the literature. The study expanded Computable General Equilibrium to Spatial Computable General Equilibrium (SCGE) and simulated the paralysis and overloading levels of transpose, internet structure.

Compared with the difference of methodology, Clower (2005) claimed the assessment of the economic impact of natural disasters is important. Analysis of disaster losses usually can be divided into macro-economic and micro-economic analysis. The former focused on a country or at least one of the state’s gross domestic product impact assessments, the latter targeted the natural disaster losses which include the dynamic comparative of inter-temporal costs at different times.

Lin et al. (2010) combined the regional and supply side Input-Output analysis developed by Miller and Blair (2009) to evaluate the losses caused by natural disasters in agricultural productions of regional economies. They found that losses of agricultural productions provoked by natural disasters damaged income and employment, and the losses accounted for about 30% of Agriculture GDP. Charlotte (2003) measured the potential impact index of disasters by macro-economic side and concluded that the potential impact of disasters can be divided into the direct costs of physical losses, indirect costs of production losses, the second round or the overall affection in short run, and the losses of long-term effect such as government budget balance.

The main advantage of Regional Input-Output model is that both direct and indirect effects can be accounted. Other methodologies are econometric economic model and computable general equilibrium (CGE). The econometric economic model needs comprehensive data for simulation, whereas the computable general equilibrium results in being more complex than Regional Input-Output model. In this paper, we shall apply side Input-Output analysis to explore the impacts of natural disasters.

## Methodology

IO, CGE, and other model methods are usually used in the literature for analysis, but the basic assumptions of these theories are different. Consider the degree of complementarity or substitution of bilateral trade in climate change for Taiwan and Mainland China, this paper applies the Inter-Country Input-Output model due to Miller and Blair (2009) to estimate the influence of agricultural product losses caused by climate change in Cross-Strait.

In the Inter-Country Input-Output table, total output can be represented in both middle and final demands, i.e.

1 $$ X=Z+F $$

with X, Z, and F being output vector, middle demand matrix, and final demand. In addition, the multi-regional trade matrix is given by

2 $$ Z=\left[\begin{array}{ccc}{Z}_{ij}^{LL}& \cdots & {Z}_{ij}^{LK}\\ {}\vdots & \ddots & \vdots \\ {}{Z}_{ij}^{KL}& \cdots & {Z}_{ij}^{KK}\end{array}\right] $$

where ${Z}_{ij}^{LL}$is the *i*th commodity produced in the *j*th industry located at the *L*th region. ${Z}_{ij}^{LK}$ denotes the outputs at the *K*th region derived from the *j*th industry and its inputs produced in the *L*th region. ${Z}_{ij}^{KL}$ and ${Z}_{ij}^{KK}$ are defined similarly to ${Z}_{ij}^{LL}$and ${Z}_{ij}^{LK}$, resp. The regional outputs are described below:

3 $$ {\displaystyle \begin{array}{c}{X}_1^L=\left[{z}_{11}^{LL}+\cdots +{z}_{1n}^{LL}\right]+\cdots +\left[{z}_{11}^{LK}+\cdots +{z}_{1n}^{LK}\right]+{F}_1^L\\ {}{X}_2^L=\left[{z}_{21}^{LL}+\cdots +{z}_{2n}^{LL}\right]+\cdots +\left[{z}_{21}^{LK}+\cdots +{z}_{2n}^{LK}\right]+{F}_2^L\\ {}\kern1.56em \vdots \kern1.44em \vdots \\ {}{X}_1^K=\left[{z}_{n1}^{LL}+\cdots +{z}_{nn}^{LL}\right]+\cdots +\left[{z}_{n1}^{LK}+\cdots +{z}_{nn}^{LK}\right]+{F}_n^L\\ {}{X}_1^K=\left[{z}_{11}^{KL}+\cdots +{z}_{1n}^{KL}\right]+\cdots +\left[{z}_{11}^{KK}+\cdots +{z}_{1n}^{KK}\right]+{F}_1^K\\ {}\vdots \\ {}{X}_2^L=\left[{z}_{21}^{KL}+\cdots +{z}_{2n}^{KL}\right]+\cdots +\left[{z}_{21}^{KK}+\cdots +{z}_{2n}^{KK}\right]+{F}_2^K\\ {}{X}_n^K=\left[{z}_{n1}^{KL}+\cdots +{z}_{nn}^{KL}\right]+\cdots +\left[{z}_{n1}^{KK}+\cdots +{z}_{nn}^{KK}\right]+{F}_n^L\end{array}} $$

If the input coefficients are fixed and the relationships of input and output are regulated, the coefficient of middle input in different regions is provided by the following expression:

4 $$ A=\left[\begin{array}{ccc}{A}^{LL}& \cdots & {A}^{LK}\\ {}\vdots & \ddots & \vdots \\ {}{A}^{KL}& \cdots & {A}^{KK}\end{array}\right]=\left[\begin{array}{ccc}\left[\raisebox{1ex}{${z}_{ij}^{LL}$}\!\left/ \!\raisebox{-1ex}{${X}_j^L$}\right.\right]& \cdots & \left[\raisebox{1ex}{${z}_{ij}^{LK}$}\!\left/ \!\raisebox{-1ex}{${X}_j^K$}\right.\right]\\ {}\vdots & \ddots & \vdots \\ {}\left[\raisebox{1ex}{${z}_{ij}^{KL}$}\!\left/ \!\raisebox{-1ex}{${X}_j^L$}\right.\right]& \cdots & \left[\raisebox{1ex}{${z}_{ij}^{KK}$}\!\left/ \!\raisebox{-1ex}{${X}_j^K$}\right.\right]\end{array}\right] $$

We also define the total output matrix, *X*, and the final demand matrix, *F*, in the following terms:

$$ X=\left[\begin{array}{c}{X}^L\\ {}\vdots \\ {}{X}^K\end{array}\right]F=\left[\begin{array}{c}{F}^L\\ {}\vdots \\ {}{F}^K\end{array}\right] $$

The equation of balance between supply and demand is given by

5 $$ X= AX+F $$

Hence,

6 $$ \left(I-A\right)X=F $$

with (*I* − *A*) being the Leontief matrix. Thus, *X* can be solved whenever such a matrix is non-singular. In fact,

7 $$ X={\left(I-A\right)}^{-1}F $$

In Eq. (7), (*I* − *A*)^−1^ is the matrix of direct and indirect requirements, also called as matrix of inter-industry interdependence coefficients or Leontief inverse matrix. Equation (7) explains the new equilibrium outputs as the final demand changes by

8 $$ \varDelta X={\left(I-A\right)}^{-1}\varDelta F $$

The Input-Output table allows classifying sections such as domestic and imported goods. Thus, the final demand can be decomposed into domestic part (*Zd*)and other part(*Zm*)to explore the dependence among different industries in the process of international trade:

9 $$ Z=\left[\begin{array}{ccc}\left[ Zd{i}_{ij}^{LL}+Z{m}_{ij}^{LL}\right]& \cdots & \left[Z{d}_{ij}^{LK}+Z{m}_{ij}^{LK}\right]\\ {}\vdots & \ddots & \vdots \\ {}\left[Z{d}_{ij}^{KL}+Z{m}_{ij}^{KL}\right]& \cdots & \left[Z{d}_{ij}^{KK}+Z{m}_{ij}^{KK}\right]\end{array}\right]={Z}^D+{Z}^M $$

10 $$ F=\left[\begin{array}{c}\left[F{d}_{ij}^L+F{m}_{ij}^L\right]\\ {}\vdots \\ {}\left[F{d}_{ij}^K+F{m}_{ij}^K\right]\end{array}\right]=\left[\begin{array}{c}\left[F{d}_{ij}^L\right]\\ {}\vdots \\ {}\left[F{d}_{ij}^K\right]\end{array}\right]+\left[\begin{array}{c}\left[F{m}_{ij}^L\right]\\ {}\vdots \\ {}\left[F{m}_{ij}^K\right]\end{array}\right]={F}^D+{F}^M $$

Hence, Eq. (4) can be rewritten as follows:

11 $$ A=\left[\begin{array}{ccc}\left[\raisebox{1ex}{$Z{d}_{ij}^{LL}+Z{m}_{ij}^{LL}$}\!\left/ \!\raisebox{-1ex}{${X}_j^L$}\right.\right]& \cdots & \left[\raisebox{1ex}{$Z{d}_{ij}^{LK}+Z{m}_{ij}^{LK}$}\!\left/ \!\raisebox{-1ex}{${X}_j^K$}\right.\right]\\ {}\vdots & \ddots & \vdots \\ {}\left[\raisebox{1ex}{$Z{d}_{ij}^{KL}+Z{m}_{ij}^{KL}$}\!\left/ \!\raisebox{-1ex}{${X}_j^L$}\right.\right]& \cdots & \left[\raisebox{1ex}{$Z{d}_{ij}^{KK}+Z{m}_{ij}^{KK}$}\!\left/ \!\raisebox{-1ex}{${X}_j^K$}\right.\right]\end{array}\right] $$

If both Eqs. (9) and (10) are substituted in Eq. (5), it holds that

12 $$ X={A}^DX+{F}^D $$

$$ {A}^D=\left[\begin{array}{ccc}\left[\raisebox{1ex}{$Z{d}_{ij}^{LL}$}\!\left/ \!\raisebox{-1ex}{${X}_j^L$}\right.\right]& \cdots & \left[\raisebox{1ex}{$Z{d}_{ij}^{LK}$}\!\left/ \!\raisebox{-1ex}{${X}_j^K$}\right.\right]\\ {}\vdots & \ddots & \vdots \\ {}\left[\raisebox{1ex}{$Z{d}_{ij}^{KL}$}\!\left/ \!\raisebox{-1ex}{${X}_j^L$}\right.\right]& \cdots & \left[\raisebox{1ex}{$Z{d}_{ij}^{KK}$}\!\left/ \!\raisebox{-1ex}{${X}_j^K$}\right.\right]\end{array}\right]{F}^D=\left[\begin{array}{c}\left[F{d}_{ij}^L\right]\\ {}\vdots \\ {}\left[F{d}_{ij}^K\right]\end{array}\right] $$

As such, the Leontief matrix can be solved in domestic goods by

13 $$ \left(I-{A}^D\right)X={F}^D $$

Let us determine *X* as well as the equation that analyses the changes of final demand:

14 $$ X={\left(I-{A}^D\right)}^{-1}{F}^D $$

15 $$ \varDelta X={\left(I-{A}^{\mathrm{D}}\right)}^{-1}\varDelta {F}^{\mathrm{D}} $$

The output effect in Eq. (15) can be transformed into the value-added effect:

16 $$ \varDelta V=v{\left(I-{A}^{\mathrm{D}}\right)}^{-1}\varDelta {F}^{\mathrm{D}} $$

From Eq. (7), both *X* = (*I* − *A*)^−1^*F* and the inverse of the Leontief matrix, (*I* − *A*)^−1^, are known. Denote *B* = (*I* − *A*)^−1^ with elements named as*b*_*ij*_. Thus, we can write

17 $$ B={\left(I-A\right)}^{-1}=\left[\begin{array}{c}{b}_{11}{b}_{12}\cdots {b}_{1i}{b}_{1j}\cdots {b}_{1n}\\ {}{b}_{21}{b}_{22}\cdots {b}_{2i}{b}_{2j}\cdots {b}_{2n}\\ {}\vdots \\ {}{b}_{i1}{b}_{i2}\cdots {b}_{ii}{b}_{ij}\cdots {b}_{\boldsymbol{in}}\\ {}{b}_{j1}{b}_{j2}\cdots {b}_{ji}{b}_{jj}\cdots {b}_{jn}\\ {}\vdots \\ {}{b}_{n1}{b}_{n2}\cdots {b}_{ni}{b}_{nj}\cdots {b}_{nn}\end{array}\right] $$

Notice that *b*_*ij*_ is the final demand-to-output multiplier with

18 $$ {b}_{jj}=\frac{\varDelta {X}_i}{\varDelta {F}_j}. $$

Let ${b}_{ij}^{\ast }$ be the ratio between *b*_*ij*_ and *b*_*jj*_, i.e.

19 $$ {b}_{ij}^{\ast }=\frac{b_{ij}}{b_{jj}} $$

Next, let us substitute Eq. (19) into Eq. (17):

20 $$ {B}^{\ast }={\left(I-{A}^{\ast}\right)}^{-1}=\left[\begin{array}{c}\frac{b_{11}}{b_{11}}\frac{b_{12}}{b_{22}}\cdots \frac{b_{1i}}{b_{ii}}\frac{b_{1j}}{b_{jj}}\cdots \frac{b_{1n}}{b_{nn}}\\ {}\frac{b_{21}}{b_{11}}\frac{b_{22}}{b_{22}}\cdots \frac{b_{2i}}{b_{ii}}\frac{b_{2j}}{b_{jj}}\cdots \frac{b_{2n}}{b_{nn}}\\ {}\vdots \\ {}\frac{b_{i1}}{b_{11}}\frac{b_{i2}}{b_{22}}\cdots \frac{b_{ii}}{b_{ii}}\frac{b_{ij}}{b_{jj}}\cdots \frac{b_{\boldsymbol{in}}}{b_{nn}}\\ {}\frac{b_{j1}}{b_{11}}\frac{b_{j2}}{b_{22}}\cdots \frac{b_{ji}}{b_{ii}}\frac{b_{jj}}{b_{jj}}\cdots \frac{b_{jn}}{b_{nn}}\\ {}\vdots \\ {}\frac{b_{n1}}{b_{11}}\frac{b_{n2}}{b_{22}}\cdots \frac{b_{ni}}{b_{ii}}\frac{b_{nj}}{b_{jj}}\cdots \frac{b_{nn}}{b_{nn}}\end{array}\right] $$

or equivalently,

21 $$ {B}^{\ast }={\left(I-{A}^{\ast}\right)}^{-1}=\left[\begin{array}{c}1\kern1.25em {b}_{12}^{\ast}\kern1.5em \cdots {b}_{1i}^{\ast}\kern0.75em {b}_{1j}^{\ast}\cdots {b}_{1n}^{\ast}\\ {}\ {b}_{21}^{\ast}\kern1.25em 1\kern1.5em \cdots {b}_{2i}^{\ast}\kern0.75em \ {b}_{2j}^{\ast}\cdots {b}_{2n}^{\ast}\\ {}\vdots \kern1.75em \\ {}\ {b}_{i1}^{\ast}\kern1em \ {b}_{i2}^{\ast}\cdots 1\kern0.75em \ {b}_{ij}^{\ast}\cdots {b}_{in}^{\ast}\\ {}\ {b}_{j1}^{\ast}\kern0.75em \ {b}_{j2}^{\ast}\cdots {b}_{ji}^{\ast}\kern0.75em 1\cdots {b}_{jn}^{\ast}\\ {}\vdots \kern2em \\ {}\ {b}_{n1}^{\ast}\kern0.75em \ {b}_{n2}^{\ast}\cdots {b}_{ni}^{\ast}\kern0.75em \ {b}_{nj}^{\ast}\kern0.75em \cdots 1\end{array}\right] $$

Similarly, the total output effect under different situations such as final demand changes, domestic goods, output effect, and the value-added effect can be calculated throughout the following expressions:

22 $$ \varDelta X={\left(I-{A}^{\ast}\right)}^{-1}\varDelta \overline{X}, $$

23 $$ \varDelta X={\left(I-{A}^{D\ast}\right)}^{-1}\varDelta \overline{X}, $$

and

24 $$ \varDelta V=v{\left(I-{A}^{D\ast}\right)}^{-1}\varDelta \overline{X} $$

## Empirical analysis

In this article, we use an Input-Output table between Taiwan and Mainland China in 2006 and 2011 involving 96 sectors (Lin 2013). Agriculture losses refer not only those from production but also from equipment. This study is focused on the production losses. Tables 1, 2, 3, and 4 display Taiwan’s losses in the periods 2005–2017 and Taiwan’s losses in the periods 2005–2017, resp. Table 1 highlights the increasing of losses year by year in Taiwan. The worst losses occurred in 2016, which caused a drawdown in supplies to several industries relying on agricultural inputs. The total losses in Taiwan from 2005 to 2017 amount to 156.6 billion. Table 1 also shows that losses caused by typhoon and heavy rain account for 83% (129.9 billion). As such, these two natural disasters caused a great impact in agriculture.

**Table 1:** **Production losses in Taiwan by disasters**. Unit: thousand NT$

Disasters	Total	2005	2006	2007	2008	2009	2010	2011	2012	2013	2014	2015	2016	2017
Hail	170,469				27,866		4128	23,819		16,517	34,031		19,204	44,904
Earthquake	25,547						12,440						13,107	
Low temperature	19,500,667	3,292,618	2,676,761	34,140	259,220	408,528	1,038,271	321,050	199,640	223	323,930	15,561	10,852,359	78,366
Strange wind	102,972			9577					8454	25,164	59,777			
High temperature	75,852					53,622						22,230		
Drought	564,535			52,250				213,986		186,534	49,998	10,670		51,097
Foehn	25,754			3753	16,105				5896					
Thunderstorm	6342						6342							
Heavy rain	18,960,784	4,282,080	4,105,562	448,346	483,135	10,189	328,523	2,393,188	101,148	385,211	800,249	537,777	2,350,066	2,735,310
Typhoon	110,953,729	11,746,564	11,406,060	10,229,757	12,031,029	10,528,637	7,091,290	193,815	4,156,646	6,418,937	1,213,440	13,382,513	21,589,713	965,328
Front	2,729,130					108,411			688,653	136,663	601,070	861,029		333,304
Tornado	69,867			2890	4925	158		886			1824	59,184		
Rain haze	3,411,433			29,266					384,490	2,312,163			685,514	
Total	156,597,081	19,321,262	18,188,383	10,809,979	12,822,280	11,109,545	8,480,994	3,146,744	5,544,927	9,481,412	3,084,319	14,888,964	35,509,963	4,208,309

Data source: Council of Agriculture (2018) and 2017 Yearly Report of Taiwan’s Agriculture Statistics

**Table 2:** **Production losses in Taiwan’s agriculture by products**. Unit: thousand NT$

Year	Total	Agriculture	Livestock	Fishery	Forest
2005	20,476,776	18,000,771	364,190	1,857,258	254,557
2006	3,250,295	3,137,993	27,734	73,100	11,468
2007	11,069,356	10,637,125	104,869	281,278	46,084
2008	13,419,717	12,559,055	117,984	719,862	22,816
2009	20,527,517	10,893,704	1,556,337	4,969,907	3,107,569
2010	9,114,662	8,069,760	192,430	817,612	34,860
2011	3,424,159	3,146,149	3171	269,381	5458
2012	5,750,631	5,545,099	17,667	142,941	44,924
2013	9,699,921	9,481,411	82,764	97,934	37,812
2014	3,124,563	3,084,320	4801	20,847	14,595
2015	16,050,399	14,432,167	25,969	246,936	60,093
2016	38,339,665	27,283,608	79,367	6,738,245	1,408,743
2017	4,313,482	3,973,372	24,234	188,660	5284

The same data source as in Table 1

**Table 3:** **Area affected by natural disasters in Mainland China.** Unit: thousand hectares

Disaster	2005	2006	2007	2008	2009	2010	2011	2012	2013	2014	2015	2016	2017
Drought	16,028	20,738	29,386	12,137	29,259	13,259	16,304	9340	14,100	12,272	10,610	9873	9,875
Flood disaster	10,932	8003	10,463	6477	7613	17,525	6863	7730	8760	4718	5620	8531	5,415
Wind hail	2977	4387	2986	4180	5493	2180	3309	2781	3387	3225	2918	2908	2,268
Low temperature and freezing	4428	4913	4072	14,696	3673	4121	4447	1618	2320	2133	900	2885	525
Typhoon	4453	2952	2086	2310	1146	342	0	0	0	0	0	0	0
Total	38,818	40,993	48,993	39,800	47,184	37,427	30,923	21,469	28,567	22,348	20,048	24,197	18,083

Data source: Ministry of Agriculture, People’s Republic of Mainland China, http://www.agri.gov.cn

**Table 4:** **Amount of losses in China by disaster**. Unit: thousand hectares, %, 100 million RMB

Year	2005	2006	2007	2008	2009	2010	2011	2012	2013	2014	2015	2016	2017
Affected area	38,818	40,993	48,993	39,800	47,184	37,427	30,923	21,469	28,567	22,348	20,048	24,197	18,083
Arable land	130,039	130,039	121,735	121,716	121,716	121,716	121,716	135,158	135,163	135,057	134,999	134,921	134,881
Ratio of disaster affection	29.85	31.52	40.25	32.7	38.77	30.75	25.41	15.88	21.14	16.55	14.85	17.93	13.41
Production													
Agriculture	19,613	21,522	24,658	28,044	30,778	36,941	41,989	46,941	51,497	54,772	57,636	59,288	61,720
Livestock	13,311	12,084	16,125	20,584	19,468	20,826	25,771	27,189	28,436	28,956	29,780	31,703	30,285
Forest	1426	1611	1862	2153	2193	2595	3121	3447	3902	4256	4436	4632	4992
Fishery	4016	3971	4458	5203	5626	6422	7568	8706	9635	10,334	10,881	11,603	12,317
Loss													
Agriculture	5855	6785	9924	9170	11,931	11,359	10,668	7456	10,884	9063	8559	10,633	8275
Livestock	3973	3809	6490	6731	7547	6404	6547	4319	6010	4791	4422	5686	4060
Forest	426	508	749	704	850	798	793	548	825	704	659	831	669
Fishery	1199	1252	1794	1701	2181	1975	1923	1383	2036	1710	1616	2081	1651

The same data source as in Table 4

Table 3 exhibits the area affected by natural disasters in Mainland China from 2005 to 2017. Notice that the area affected increases year by year, with drought being the worst disaster in Mainland China. If the affected areas are divided by total arable lands, the amount of losses each year can be calculated. In this way, Table 4 states that the economic losses vary from 114.5 to 192.3 billion each year.

## Economic impact analysis

Supply-side Inter-Country Input-Output analysis is applied to assess the impact caused by natural disasters in both Taiwan and China’s economic systems. Losses were estimated in value-added effects. Table 5 provides the value-added losses provoked by natural disasters in Taiwan in the periods 2005–2017. Those effects range from 6 billion to 39 billion NT. The worst reduction was found in agriculture accounting for 94.6% of total losses, followed by wholesale and retail trade industries. The last place corresponds to chemical fertilizer industry.

**Table 5:** **Losses in value-added in Taiwan by natural disasters**. **Unit: Million NT**

Sectors		2005		2006		2007		2008		2009		2010		2011		2012		2013		2014		2015		2016		2017	
		GDP	Rank	GDP	Rank	GDP	Rank	GDP	Rank	GDP	Rank	GDP	Rank	GDP	Rank	GDP	Rank	GDP	Rank	GDP	Rank	GDP	Rank	GDP	Rank	GDP	Rank
		Effect		Effect		Effect		Effect		Effect		Effect		Effect		Effect		Effect		Effect		Effect		Effect		Effect	
1	Farming products	46,012	1	7996	1	27,110	1	32,035	1	28,394	1	20,630	1	4080	1	7190	1	12,295	1	3999	1	18,713	1	35,393	1	5152	1
2	Livestock products	602	6	53	8	194	8	223	8	2292	4	311	5	5	17	12	13	37	10	5	16	25	16	56	16	12	11
3	Forest products	778	5	37	11	147	9	79	15	9305	3	112	10	9	11	74	5	63	5	25	4	100	5	2303	4	9	14
4	Fishery products	3710	2	147	4	567	3	1441	3	9913	2	1633	2	194	3	103	4	71	4	15	7	178	4	4859	2	136	4
5	Agricultural services	2357	3	403	2	1366	2	1621	2	1648	5	1056	3	329	2	577	2	988	2	320	2	1500	2	2905	3	415	2
6	Crude petroleum and natural gas extraction	186	13	30	13	101	13	123	12	180	15	83	13									1	68	5	51		
8	Other non-metallic minerals	249	11	42	9	142	10	169	10	197	14	111	11					1	53			1	52	3	56		
13	Animal feeds	112	16							288	11	52	16	3	20	3	26	5	25	1	33	6	28	64	15	3	23
27	Petrochemical raw materials and petroleum refining products	171	14	24	14	83	14	106	13	211	13	76	14	11	9	16	11	26	12	8	11	40	11	156	9	12	10
29	Basic chemical materials			15	19	51	19	61	19					6	15	10	16	17	16	5	15	25	15	50	17	7	16
30	Chemical fertilizers	527	7	91	5	308	5	365	5	357	8	236	6	19	5	34	6	58	6	19	5	89	6	168	7	24	5
33	Pesticides and herbicides	140	15	24	15	81	15	96	14	107	20	62	15	11	10	19	10	33	11	11	10	50	10	94	13	14	9
39	Plastic products	109	17	18	16	62	16	74	16			49	17	5	16	9	17	16	17	5	17	24	17	49	18	7	17
66	Electricity supply	363	9	57	7	195	7	238	7	345	9	162	8	7	14	11	15	18	15	6	14	27	14	92	14	8	15
71	Wholesale trade and retail trade	1134	4	165	3	565	4	705	4	1372	6	511	4	121	4	189	3	318	3	99	3	477	3	1440	5	144	3
72	Railroad vehicle transportation and land transportation	267	10	41	10	139	11	170	9	311	10	118	9	8	12	13	12	22	13	7	12	33	12	107	11	10	12
83	Telecommunication services	108	18	16	17	56	17	69	17	121	18	48	19	3	21	4	22	7	22	2	22	11	21	37	24	3	21
85	Finance, securities, futures, and other activities auxiliary to financial service activities	429	8	66	6	225	6	275	6	495	7	191	7	19	6	32	7	54	7	17	6	81	7	214	6	23	6
87	Real estate services	108	19	16	18	54	18	67	18	131	17	48	18	8	13	11	14	18	14	6	13	28	13	99	12	9	13
89	Professional, scientific, and technologic services	223	12	35	12	119	12	145	11	233	12	100	12	15	7	25	8	43	8	13	8	64	8	162	8	19	7
95	Public administration and social association services	95	20	15	20	51	20	61	20	135	16	41	20	12	8	22	9	37	9	12	9	56	9	146	10	15	8
96	Repair, domestic, and personal services									111	19			3	23	4	21	7	21	2	21	11	22	38	23	3	22
	Total	59,140		9,504		32,347		39,038		57,817		26,279		4913		8428		14,245		4614		21,709		48,999		6078	

Following the above, wholesale and retail industries appear in second place with an input coefficient equal to 0.07 units in agricultural production offer. Since the forward linkage of wholesale and retail is bigger, the economic effect of that industry accounts for 0.5% averagely.

The thirdly ranked flow coefficient corresponds to chemical fertilizers with 0.10 units in agricultural production offer. A large backward linkage with agriculture leads to production losses as well as losses in value-added effects of chemical fertilizers industry.

Table 6 displays the value-added losses by natural disasters in Mainland China from 2005 to 2017. The range of effect lies between 1965.6 to 5935.4 billion RMB. Agriculture accounts the largest effect by 87.4% in 2017, followed by wholesale and retail trade, and animal feed.

**Table 6:** Losses in value-added by natural disasters in China. Unit: 100Million RMB

96 sectors Cross-Strait Inter-country I-O table		2005		2006		2007		2008		2009		2010		2011		2012		2013		2014		2015		2016		2017	
		GDP	Rank	GDP	Rank	GDP	Rank	GDP	Rank	GDP	Rank	GDP	Rank	GDP	Rank	GDP	Rank	GDP	Rank	GDP	Rank	GDP	Rank	GDP	Rank	GDP	Rank
		Effect		Effect		Effect		Effect		Effect		Effect		Effect		Effect		Effect		Effect		Effect		Effect		Effect	
1	Farming products	15,657	1	18,001	1	26,480	1	24,595	1	31,795	1	24,784	1	15,002	1	10,445	1	15,201	1	12,623	1	11,904	1	14,827	1	11,482	1
2	Livestock products	5668	2	5440	2	9258	2	9596	2	10,769	2	7520	2	6355	2	4194	2	5837	2	4655	2	4297	2	5524	2	3,947	2
3	Forest products	1281	4	1528	4	2255	4	2119	4	2560	4	1976	4	1058	4	731	4	1101	4	939	4	879	4	1108	4	892	4
4	Fishery products	2417	3	2522	3	3622	3	3439	3	4401	3	3275	3	2395	3	1719	3	2527	3	2120	3	2002	3	2578	3	2,041	3
5	Agricultural services	1014	6	1125	6	1700	6	1615	6	2028	6	1548	6	113	14	79	14	115	14	96	14	90	14	114	14	89	13
6	Crude petroleum and natural gas extraction	86	15	95	15	142	15	135	17	169	15	129	15	143	11	99	11	144	11	119	11	112	11	141	11	108	11
8	Other non-metallic minerals	104	13	117	13	175	13	165	13	209	13	160	13	185	10	128	10	186	10	154	10	144	10	181	10	139	10
11	Flour	82	17	81	17	134	17	137	16	157	17	112	17	79	18	53	19	74	19	60	19	55	19	71	19	52	19
13	Animal feeds	318	7	310	8	515	7	527	7	603	7	425	8	356	7	239	7	336	7	271	7	251	7	323	7	236	8
27	Petrochemical raw materials and petroleum refining products	91	14	98	14	148	14	142	14	177	14	133	14	111	15	77	15	111	15	92	15	87	15	109	15	84	15
30	Chemical fertilizers	194	9	221	9	328	9	307	9	393	9	304	9	317	8	220	8	320	8	265	8	250	8	312	8	241	7
33	Pesticides and herbicides			60	20	89	20			106	20	82	20	77	19	53	18	78	18	65	18	61	18	76	18	59	18
66	Electricity supply	188	10	204	10	311	10	299	10	370	10	279	10	202	9	140	9	202	9	167	9	157	9	197	9	151	9
71	Wholesale trade and retail trade	1109	5	1133	5	1814	5	1809	5	2138	5	1553	5	449	5	307	5	439	5	360	5	336	5	426	5	321	5
72	Railroad vehicle transportation and land transportation	168	11	180	11	277	11	269	11	329	11	245	11	161	13	110	13	158	13	130	13	122	13	154	13	117	14
83	Telecommunication services	63	19	68	19	104	19	100	19	123	19	92	19	29	27	20	27	29	27	24	27	22	26	28	27	22	26
85	Finance, securities, futures, and other activities auxiliary to financial service activities	297	8	314	7	490	8	479	8	579	8	429	7	372	6	256	6	370	6	306	6	287	6	361	6	276	6
87	Real estate services	86	16	89	16	140	16	138	15	165	16	121	16	66	20	46	20	66	20	54	20	51	20	64	20	49	20
89	Professional, scientific, and technologic services	157	12	165	12	259	12	254	12	306	12	226	12	133	12	91	12	132	12	109	12	102	12	128	12	98	12
95	Public administration and social association services	66	18	71	18	111	18	108	18	130	18	97	18	9	54	6	54	9	54	7	54	7	54	9	54	7	54
96	Repair, domestic, and personal services	53	20					85	20					37	24	25	23	37	23	30	23	28	23	36	23	27	23
	Total	30,047		32,831		49,908		47,829		59,354		44,871		28,545		19656		28,364		23,381		21,936		27,638		21,102	

Agriculture has the greatest impact by natural disasters. An input coefficient of agriculture equal to 1.14 means that losses will bring damage forward linkage. It holds that losses of productions and value-added in agriculture account for more than 65% jointly.

Agriculture, wholesale and retail trade, animal feed, and chemical fertilizer industries present the highest impacts between Taiwan and Mainland China. Comparing both Taiwan’s and China’s multipliers, we found that the Chinese one (2.6) stands larger than Taiwan’s (2.9). This result highlights that Taiwan’s losses are larger than Chinese ones whenever the same natural disasters are faced in Cross-Strait.

## Conclusions

To estimate direct and indirect losses caused by natural disasters, this study applies Inter-Country Input-Output (ICIO) analysis developed by Miller and Blair (2009) to discuss the development among Cross-Strait industries as they face disaster losses. In this study, we applied Supply-side Inter-Country Input-Output analysis with the aim to assess the impact of climate change in Taiwan and China’s economic systems. Our main results are stated below:

- The value-added losses caused by natural disasters mainly involve agriculture, forestry, fishery, wholesale and retail trade, animal feed, and chemical fertilizer industries. These sectors account for 87.4% in Mainland China and 94.6% in Taiwan of total separately.
- The impacts caused by other natural disasters provoke agricultural losses which, in turn, influence other industries via industrial linkage. Our empirical results suggest a multiplier for agricultural losses in Mainland China (2.6) smaller than the corresponding one for agricultural losses in Taiwan (2.9).

Considering the economic development trend, Taiwan Province, Japan, and South Korea have certain similarities; however, this study does not furtherly extend to study other relate issues. Further study will apply the methodology to estimate other country’s economic behaviour.

## Appendix. The compiling explanation of 96 sectors Cross-Strait ICIO table

National Input-Output tables are able to track domestic value-added flows as they pass through the producers of a given economy, but they are not able to identify where the value-added in imports originated. To fully account for value-added flows therefore, and to construct measures of trade in value-added, an Inter-Country Input-Output (ICIO) table is needed.

ICIO tables are the underpinnings of trade measuring in value-added. There is a consensus among international statistical agencies that direct measuring of trade in value-added is extremely difficult. The most feasible and promising approach to develop comprehensive and consistent measure for trade in value-added that goes beyond case studies of individual products (such as the iPod) has to use ICIO tables. With a growing recognition of the need to have a comprehensive global I-O table in order to measure trade in value-added properly and timely, four ICIO databases have been developed. The most widely known ICIO databases being used to discuss economic and trade issue are The World Input-Output (WIOD) database, GTAP database, The OECD ICIO database, and IDE-JETRO Asia International Input-Output tables. The common problem for these ICIO databases is that the classification of sectors is too aggregated. Therefore, in this study, apply Lin (2013) compile the 96 sectors Cross-Strait Inter-country Input Output (ICIO) table in 2006.

### The methods and steps of compiling explanation of 96 sectors Cross-Strait ICIO table

The source of Taiwan-Mainland China ICIO table came from 166 sectors Taiwan Input Output table in 2006 compiling by Directorate-General Budget, Accounting and Statistics (DGBAS), Executive Yuan of Taiwan, and 135 sectors Mainland China Input Output table in 2007 compiling by National Bureau of Statistics of Mainland China.

#### Integrating the classification sectors of Taiwan and Mainland China Input Output tables

The classification sectors of Taiwan Input Output table in 2006 and Mainland China Input Output table in 2007 are not the same. The author integrated the classification sectors of 166 sectors in Taiwan and 135 sectors in Mainland China Input Output table to 96 sectors (see Table 7)

**Table 7:** The sector classification of 96 Sectors Cross-Strait International Input-Output Table in 2006

No.	Sectors	No.	Sectors
001	Farming products	049	Audio and video electronic products
002	Livestock products	050	Other electronic equipment and unrecorded data storage media
003	Forest products	051	Precision instruments and apparatus
004	Fishery products	052	Power generation, transmission, and distribution machinery
005	Agricultural services	053	Wires, cables, and wiring devices
006	Crude petroleum and natural gas extraction	054	Domestic appliances
007	Metallic minerals	055	Other electrical materials
008	Other non-metallic minerals	056	Metal processing machinery
009	Slaughtering and by-products	057	Other special-purpose machinery
010	Edible oil & fat by-products	058	Boilers and pressure containers
011	Flour	059	General-purpose machinery, repair, and installation of industrial machinery and equipment
012	Sugar	060	Motor vehicles
013	Animal feeds	061	Ships
014	Seasonings	062	Other transport equipment
015	Seasonings	063	Furniture
016	Other foods	064	Education and entertainment articles
017	Alcoholic beverages	065	Other manufactures
018	Non-alcoholic beverages	066	Electricity supply
019	Tobacco	067	Gas supply
020	Fibre products	068	City water and remediation services
021	Wearing apparel and clothing accessories	069	Construction
022	Leather and other leather products	070	Wholesale trade and retail trade
023	Wood products	071	Railroad vehicle transportation and land transportation
024	Pulp, paper, and paper products	072	Water transportation
025	Printing and reproduction of recorded media	073	Air transportation
026	Petrochemical raw materials and petroleum refining products	074	Other transportation and supporting services to transportation
027	Coke and other coal products	075	Warehousing and storage
028	Basic chemical materials	076	Postal and courier services
029	Chemical fertilizers	083	Finance, securities, futures and other activities auxiliary to financial service activities
030	Compound fertilizers	084	Insurance
031	Chemical fibres	085	Real estate services
032	Pesticides and herbicides	077	Accommodation services
033	Coatings, dyes, and pigments	078	Food and beverage services
034	Cleaning preparations and cosmetics	079	News and publishing
035	Other chemical products	080	Communication services
036	Medicines	081	Telecommunication services
037	Rubber products	082	Information services
038	Plastic products	086	Research and development services
039	Glass and glass products	087	Professional, scientific, and technologic services
040	Ceramic products	088	Renting and leasing services
041	Cement and other non-metallic mineral products	089	Travel agency services
042	Pig iron and crude steel	090	Public facilities, buildings, and greenery services
043	Primary iron and steel products	091	Educational services
044	Other metals	092	Medical, health, and social services
045	Fabricated metal products	093	Arts, entertainment, and recreation services
046	Electronic components and parts	094	Public administration and social association services
047	Computer products and computer peripheral equipment	095	Repair, domestic, and personal services
048	Communication equipment	096	Undistributed and waste

#### Estimating Mainland China domestic and import Input Output tables

There are 166 sectors’ Taiwan domestic and import Input Output tables in 2006 compiling by DGBAS of Taiwan. In Mainland China, there is only an Input Output table at producers’ prices in 2007. Therefore, the author applied the assumption of all industries using the assumption of import rate of domestic demand (including output and net import) which is equal to estimate the 135 sectors of Mainland China domestic and import Input Output tables in 2007.

#### Adjustment the base of pricing

Choose 2006 as the benchmark year. Then, calculate the output, import, salary, and depreciation price deflators to deflate the 96 sectors in Mainland China Input Output table from 2006 to 2007. It means 2006 is the benchmark year. The next step is applying the exchange rate to adjust the RMB to NT pricing.

#### Using source and usage of Cross-Strait trade data to estimate the ICIO table

Finally, use the source of Cross-Strait export and import statistic data to separate the trade data in original Input Output table. Then, use the trade data to estimate the intermediate and final demand of ICIO table. Then, adjust the balance of Cross-Strait ICIO table between supply and demand sides.

### The fragment and formulas of 96 sectors Cross-Strait ICIO table

Following the above steps could finish compiling 96 sectors Cross-Strait ICIO table in 2006. The fragment and relationship of vectors and matrixes in Cross-Strait ICIO table can be seen in Table 8.

**Table 8:** Framework of Cross-Strait Inter-country Input Output Table

Supply			Demand								
			Intermediate demand (X)		Final demand (F)					Total supply	
			(Taiwan)1,2, …, j,…,n	(Mainland China)1,2,…, j,…,n	(Taiwan)(*F*^*T*^)	(Mainland China)(*F*^*C*^)	Export(E)			Output (X)	Import (M)
							(Taiwan)	(Mainland China)	Rest of the World(R)		
Intermediate input	(Taiwan)1,2,…, i,…,n	D	${Z_{ij}^D}^{TT}$		${F_i^D}^{TT}$			${E}_i^{TC}$	${E}_i^{TR}$	${X}_i^T$	
		M		${Z_{ij}^M}^{TC}$		${F_i^M}^{TC}$					${M}_i^{S_1C}$
	(Mainland China)1,2,…, i,…,n	D		${Z_{ij}^D}^{CC}$		${F_i^D}^{CC}$	${E}_i^{CT}$		${E}_i^{CR}$	${X}_i^C$	
		M	${Z_{ij}^M}^{CT}$		${F_i^M}^{CT}$						${M}_i^{S_2T}$
Rest of the world (R) 1,2,…, i,…,n			${Z_{ij}^M}^{RT}$	${Z_{ij}^M}^{RC}$	${F_i^M}^{RT}$	${F_i^M}^{RC}$					
International shipping(FI)			$F{I}_j^{ZT}$	$F{I}_j^{ZC}$	$F{I}_j^{\boldsymbol{FT}}$	$F{I}_j^{FC}$					
Primary inputs (Value-added)(V)			${V}_j^T$	${V}_j^C$							
Adjustment(A)			${A}_j^{ZT}$	${A}_j^{ZC}$	${A}_j^{\boldsymbol{FT}}$	${A}_j^{FC}$					
Total input (X)			${X}_j^T$	${X}_j^C$							

Source:Lin (2013)

${Z_{ij}^D}^{TT}$:the *j*th industry of Taiwan (T) uses the *i*th’s domestic products or service of Taiwan (T) as intermediate input

${Z_{ij}^M}^{TC}$:the *j*th industry of Mainland China (C) uses the imported products or service from the *i*th’s industry in Taiwan (T) as intermediate input

${Z_{ij}^D}^{CC}$:the *j*th industry of Mainland China (C) uses the *i*th’s domestic products or service of Mainland China (C) as intermediate input

${Z_{ij}^M}^{CT}$:the *j*th industry of Taiwan (T) uses the *i*th’s import products or service of Mainland China (C) as intermediate input

${Z_{ij}^M}^{RT}$:the *j*th industry of Taiwan (T) uses the *i*th’s import products or service of other countries (R) (except Cross-Strait) as intermediate input

${Z_{ij}^M}^{RC}$:the *j*th industry of Mainland China (C) uses the *i*th’s import products or service of other countries(R) (except Cross-Strait) as intermediate input

${F_i^D}^{TT}$:the *j*th industry of Taiwan (T) uses the *i*th’s domestic products or service of Taiwan (T) as domestic final demand

${F_i^M}^{TC}$:the *j*th industry of Mainland China (C) uses the *i*th’s import products or service of Taiwan (T) as domestic final demand

${F_i^D}^{CC}$:the *j*th industry of Mainland China (C) uses the *i*th’s domestic products or service of Mainland China (C) as domestic final demand

${F_i^M}^{CT}$:the *j*th industry of Taiwan (T) uses the *i*th’s import products or service of Mainland China (C) as domestic final demand

${F_i^M}^{RT}$:The *j*th industry of Taiwan (T) uses the *i*th’s import products or service of other countries (R) (except Cross-Strait) as domestic final demand

${F_i^M}^{RC}$:The jth industry of Mainland China (C) uses the ith’s import products or service of other countries (R) (except Cross-Strait) as domestic final demand

${E}_i^{TC}$:The *i*th industry products or service of Taiwan (T) export to Mainland China (C)

${E}_i^{TR}$:the *i*th industry products or service of Taiwan (T) export to other countries (R)

${E}_i^{CT}$:the *i*th industry products or service of Mainland China (C) export to Taiwan (T)

${E}_i^{CR}$:the *i*th industry products or service of Mainland China (C) export to other countries (R)

${X}_i^T$:the total output of Taiwan (T) *i*th industry products or service

${X}_i^C$:the total output of Mainland China (C) *i*th industry products or service

${M}_i^{S_1C}$:the *i*th industry import products or service of Mainland China (C) (S_1_:from Taiwan (T), from other countries (R)

${M}_i^{S_2T}$:the *i*th industry import products or service Taiwan (T) (S_1_:from Mainland China (C), from other countries (R)

$F{I}_j^{ZT}$:the international shipping fee and assurance of Taiwan (T) *j*th industry use import products as intermediate input

$F{I}_j^{ZC}$:the international shipping fee and assurance of Mainland China (C) *j*th industry use import products as intermediate input

$F{I}_j^{\boldsymbol{FT}}$:the international shipping fee and assurance of Taiwan (T) *j*th industry use import products as domestic final demand

$F{I}_j^{FC}$:The international shipping fee and assurance of Mainland China (C) *j*th industry use import products as domestic final demand

${A}_j^{ZT}$:the adjustment of Taiwan (T) *j*th industry use as intermediate input

${A}_j^{ZC}$:the adjustment of Mainland China (C) *j*th industry use as intermediate input

${A}_j^{\boldsymbol{FT}}$:the adjustment of Taiwan (T) *j*th industry use as domestic final demand

${A}_j^{FC}$:the adjustment of Mainland China (C) *j*th industry use as domestic final demand

${V}_j^T$:the primary input of Taiwan (T) *j*th industry

${V}_j^C$:the primary input of Mainland China (C) *j*th industry

${X}_j^T$:the total input of Taiwan (T) *j*th industry

${X}_j^C$:the total input of Mainland China (C) *j*th industry
